# Skeletal and Dental Effects of Forsus Fatigue Resistance Device Versus Twin Block Appliance for Class II Malocclusion Treatment in Growing Patients: A Systematic Review

**DOI:** 10.1002/cre2.70054

**Published:** 2024-12-12

**Authors:** Bahaa Aldeen Jeha, Rania Haddad

**Affiliations:** ^1^ Department of Orthodontics University of Damascus Dental School Damascus Syria

**Keywords:** dental, forsus, skeletal, twinblock

## Abstract

**Objective:**

This study systematically searched the literature and assessed the available evidence to compare the efficacy of Forsus Fatigue Resistance Device (FRD) versus Twin Block Appliance (TBA) in treating class II malocclusion.

**Material and Methods:**

The search for published literature was published up to May 28, 2024. The databases were included in the search: MEDLINE, PubMed, EMBASE, Cochrane Oral Trials Register, Tripe, Web of Science, and Scopus. Additionally, unpublished literature was searched on ClinicalTrials.gov, the National Research Register, and ProQuest Dissertations and Theses. All eligible studies were carefully reviewed and two reviewers independently extracted data. In cases of disagreement, an arbiter was consulted for resolution.

**Results:**

Two randomized controlled trials (RCTs) and five non‐RCTs were included in this review. The total number of patients included in the studies examining SNA, SNB, and ANB was 254. The studies also looked at the variables Go‐Gn, L1‐ML, and U1‐SN, with 279, 205, and 277 patients included for each variable, respectively. According to the evidence reported, TBA showed greater skeletal effects in terms of mandibular length and advancement. The pooled estimate revealed a statistically significant 1.3° increase in the SNB, and a decrease of −1.34° in the ANB angles for patients treated with TBA compared with those treated with FRD, with no statistically significant differences in the SNA angle. Most studies had a moderate risk of bias, while only two studies had a high risk of bias.

**Conclusion:**

FRD has been proven to be an effective treatment device for correcting ANB and restricting SNA angle, similar to TBA. However, TBA appears to offer better mandibular length and SNB outcomes.

## 1 Introduction

Around one‐third of the population experiences different severity levels of class II malocclusion (Kelly, Sanchez, and Van Kirk 1973; McLain and Proffitt 1985), which can be attributed to three specific skeletal situations, the significant factor of which is mandibular retrognathia (Clark 2014; McNamara 1981). The main treatment options available for managing skeletal defects are growth modification, dental camouflage, and orthognathic surgery. The choice of treatment depends on the patient's skeletal age, the severity of malocclusion, and their personal preferences (Patient's tolerance, knowledge, and adherence) (Clark 2014). A wide range of functional appliances are available to correct dental and/or skeletal class II malocclusion (McNamara 1981). The basic option is to use Functional devices, which fall into two main groups: fixed and removable, with the former existing as flexible, hybrid, or rigid (Papadopoulos and Elsevier 2006; Zentner 2006). Using such appliances may encourage the growth of the mandible while restraining the maxilla (Barnett et al. 2008). Orthodontic practitioners should be aware of these considerations when choosing the most suitable treatment device for their patients (Perinetti et al. 2015). The most extensively used appliances, namely the Twin Block Appliance (TBA) and Forsus Fatigue Resistance Devise (FRD), have been the focus of this review as being abundantly found in literature and clinics, with more emphasis on the former over the past recent years (Clark 2014). Although the TBA involves laboratory procedures, it has proven to be effective in correcting mandibular deformities, stimulating growth, and causing minimal dentoalveolar adverse effects (Brunharo et al. 2011; Mills and McCulloch 1998). FRD is, contrastively, a premium treatment option to promote the advancement of the mandible where a minimum amount of growth is detected in the child's puberty spurt. But with the drawback of low control of lower incisors’ inclination urges some practitioners to reinforce the FRD with either mini plates or mini‐screws to enhance treatment effects. Here, Vogt advocates FRD's clinical superiority as a “telescoping system” of a semi‐rigid nature whose coil spring is made of high‐quality nickel‐titanium (Vogt 2006). FRD can be utilized along with fixed appliances and does not need laboratory procedures. FRD does not seem to take complicated laboratory procedures, whereas TBA necessitates the use of adherents and extra lab steps. Still, the latter has long yielded considerably more tolerance and acceptance among patients than other removable appliances (Clark 1982; Jena, Duggal, and Parkash 2006).

Multiple systematic reviews and meta‐analysis have evaluated the effectiveness of the Fixed Functional Appliance (FFA) in comparison to other functional appliances (Pacha, Fleming, and Johal 2016). Some of these studies have investigated each appliance separately (Papadopoulos and Elsevier 2006; Linjawi and Abbassy 2018; Li et al. 2023), while others have conducted comparative analyses of different devices, such as the Forsus device with Herbst (Matthaios et al. 2022)and class II elastics (Matthaios et al. 2022; Janson et al. 2013). In addition, some reviews have specifically focused on the efficacy of the fixed device alone (Linjawi and Abbassy 2018), while others investigated the potential benefits of combining it with skeletal anchorage (Arvind and Jain 2021; Bakdach and Hadad 2021). All these studies aimed to provide a comprehensive evaluation of the various functional appliances used in orthodontic treatments and their effectiveness in correcting class II malocclusion. After conducting a scrutiny examination and review of the medical literature, it was found that there is no existing comparison between the two devices. As a result, the goal of this review was to address the following question: Which is a more effective treatment for class II malocclusion, Twin Block OR Forsus™ Fatigue Resistance Device?

## 2 Materials and Methods

### 2.1 Protocol and Registration

The protocol of this systemic review was registered in the PROSPERO international database, endorsed by the National Institute for Health Research, with an ID serialized as CRD42022353211. This systemic review followed the guidelines of the Preferred Reporting Items for Systematic Reviews and Meta‐Analyses (PRISMA) statement (Higgins et al. 2019) and the instructions of the Cochrane Handbook (Higgins and Green 2008). It builds on a set of criteria for including or excluding the contacted literature.

### 2.2 Human Ethical Approval and Consent to Participate

Ethical approval was not required for this systematic review as it was not applicable.

### 2.3 Informed Consent

Informed consent was not required for this systematic review as it was not applicable.

*Inclusion criteria:* Only studies comparing (TBA) with (FRD) Devices were included, the study was only included in this review if it compares TBA with FRD, irrespective of following the design of applying the research on experimental and control groups. The criteria followed the PICOS approach.

*Participants:* They should be growing patients undergoing a growth modification treatment for class II malocclusion caused by a retrognathic mandible.

*Interventions:* The study focuses on the use of FRD to correct skeletal class II malocclusion in growing patients.

*Comparison:* Made with the use of TBA as a standard functional appliance in treating retrognathic mandibular in skeletal class II growing patients.

*Outcome:* The outcomes should evaluate the skeletal (SNA, SNB, and ANB angle, Go‐Gn liner measurement) and dentoalveolar (U_1_/SN and L_1_/ML angle) effects of both TBA and FRD.

*Study design:* Both randomized control trials (RCTs) and non‐randomized control trials (Non‐RCTs) are included in this review.

*Exclusion criteria* **:** Animal studies, in vitro studies, case reports, case series, reviews, cross‐sectional studies, trials containing craniofacial syndrome, trials involving other types of appliances, and studies not matching the criteria of this systematic review were all excluded.

### 2.4 Data Collection and Management

Two reviewers, B.J. and R.H., independently extracted data using data collection forms, screened electronic citations using Rayyan® software (Baka and Fidanboy 2021), and conducted searches in published and unpublished studies. The searches covered multiple databases for published literature and specific sources for unpublished literature of search strategy and procedure are fully described in Table S1. Following this process of filtration, data from the studies were extracted independently by the two researchers and arranged into tables.

*Risk of bias (RoB) assessment:* Cochrane's RoB‐2 tool for RCTs and the ROBINS‐I tool for non‐RCTs in orthodontic interventions were used. Both reviewers conducted the assessment independently, and a third arbiter was consulted in case of disagreement. The overall risk of bias for each study was reported as low, moderate, or serious. The assessment criteria are explained below:

− Low: All domains are at low risk of bias.
− Some concerns: At least one or more domains show some concerns of bias.
− High: At least one domain is at high risk of bias.

## 3 Results

### 3.1 Literature Search Flow

**Figure 1:**
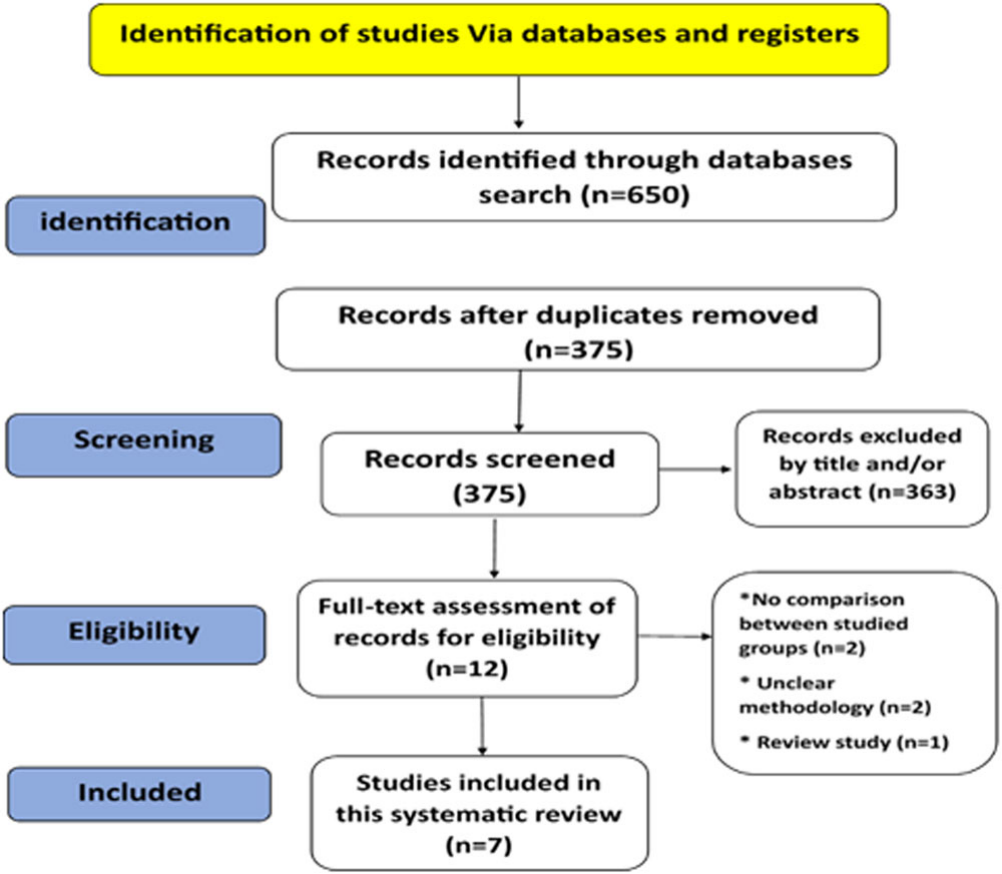
PRISMA flow diagram of the included studies.

The literature review began with an electronic search, which initially identified 650 records. After removing duplicates and irrelevant articles, we scrutinized the abstracts and full texts of the remaining records, resulting in 363 studies being disregarded. We then eliminated duplicates and articles that fell outside the defined scope (PICOS), leaving 12 studies for thorough analysis to determine their eligibility for inclusion in this systematic review. Ultimately, seven studies met the inclusion criteria and were included in the review (Hanoun et al. 2014; Yavan et al. 2021; Alhammadi et al. 2019; Tarvade et al. 2014; DiBiase, Cobourne, and Lee 2015; Ngan and Tai 2023; Antonarakis and Kiliaridis 2007). Figure 1 pinpoints this PRISMA flow procedure. Excluded studies after full‐text assessment and with reasons supporting their exclusion decision.

### 3.2 Characteristics of the Included Studies

The seven included studies were published between 2014 and 2021, and all of them were written in the English language. Five of them are non‐RCTs (Baka and Fidanboy 2021; Giuntini et al. 2015; Gulec and Goymen 2018; Hanoun et al. 2014; Yavan et al. 2021) and two are RCTs (Alhammadi et al. 2019; Tarvade et al. 2014); the sample size in each one of them ranges between 12 and 37 in the TBA group and 12–36 in the FRD ones. Moreover, the mean age of the participant included in these seven studies ranged from 11.2–14.8 years in the TBA group and 12.3–15.1 in their matching counterparts subjected to investigation in the FRD groups. All of the selected studies inspect the effect of both TBA and Fatigue Resistant Device when administering treatment to control and alleviate class II malocclusion. Nonetheless, this very consideration has been either the main outcome of the scrutinized studies or the secondary outcome to be considered by the researchers of the specific study. The characteristics of the seven selected studies are summarized in Table 1.

**Table 1:** The characteristics of included studies.

Authors, study design, year, country, reference	Title	Participants (sample size, Male/Female, Age)	Intervention	End of evaluation	Outcomes measures	Main finding
Giuntini et al. (2015). CCT, 2015, Italy	Treatment effects produced by the twin–block appliance versus the Forsus Fatigue Resistant Device in growing Class II Patients FRD* until reach edge‐to‐edge relationship.	− 91 patients divided into three group − TB (*n* = 28/19f‐9M A:12.4Y) − FRD (*n* = 36/16f–20M/A:12.3Y) ‐ CG (*n* = 27/13F‐14M/A:12.2Y).T2: post appliance remove	IG1:TB IG2:FRD G3:controL T2: at the end of comprehensive treatment with fixed appliance	T1: for all treated subjects at the beginning of orthodontic treatment T2: at the end of comprehensive treatment with fixed appliance	Skeletal Dento‐alveolar	TB produces a Greater skeletal effect than FDR in terms of mandibular advancement and growth stimulation Class II correction was more dentoalveolar than was the TB with a large amount of proclination of the mandibular incisor
Hanoun et al. (2014). CCT, 2014, Saudi Arabia	A comparison of the treatment effects of the Forsus Fatigue Resistance Device and the Twin Block appliance in patients with Class II malocclusions	92 patients divided into three groups. − TB (*n* = 37/24f–13M/A:11.2 ± 1.6Y) − FDR (*n* = 30/12f–13M/A:12.9 ± 1.1Y) − CG (*n* = 25/12f–13M/11.9 ± 1.9Y)	IG1 = TB IG2 = FRD G3 = control	T1: immediately before the insertion of FDR and before any treatment of TB T2: post appliance removes	Skeletal Dento‐alveolar	Both FDR and TB induced maxillary and mandibular dentoalveolar change but skeletal change was induced only by TB
Travade et al. (2014). RCT, 2013, India,	Dentoskeletal comparison of changes seen in class II treated by Twin bock and Forsus	20 Patients divided into two groups: TB (*n* = 10/13–17 Y) FRD (*n*10/13–17Y)	G1 = TB IG2 = FRD	T0 = before treatment T1= end of treatment	Skeletal Dento‐alveolar	TB has more mandibular lengthening effect as compared to FRD and thus it was more effective in the treatment of class II
Baka and Fidanboy (2021). CCT, 2021, Turkey	Pharyngeal airway, hyoid bone, and soft palate changes after class II treatment with Twin block and Forsus appliances during the post peak growth period	24 patients divided into two groups: − TB (*n* = 21/13F–8M/A;15.1 ± 1.8Y) − FRD (*n* = 21/16F–5M/A:14.8 ± 2.1Y).	IG_1_ = TB IG_2_ = FRD	T_0 _= before treatment T_1_ = end of treatment	Skeletal Dento‐alveolar Pharyngeal airway hyoid	FDR: caused a greater protrusion of the mandibular incisor
Yavan et al. (2021). CCT, 2021, Turkey	Comparison of Twin block appliance and Forsus Fatigue Resistant device therapies on uvulo‐glossopharyngeal dimensions: A retrospective study	50 patients divided into two groups: − FRD (*n* = 25/14f–11M/A:13.5 ± 2.8Y). TB (*n* = 25/17 f 8 M/12.5 ± 0.4Y).	IG_1_ = TB IG_2_ = FRD	T_2_: TB* until reach to class I canine relationship. FRD* until reach edge to edge relationship	Skeletal Dento‐alveolar Pharyngeal airway Uvula‐hyoid‐Tongue	TB produces more advancement in SNB angle in comparison with FRD
Alhammadi et al. (2019). RCT, 2019, Saudi Arabia	Three –dimensional skeletal and pharyngeal airway changes following therapy with functional appliance in growing skeletal class II malocclusion patients	62 female patients were divided into three groups. TB (*n* = 23/A:11.89 ± 1.85) FRD (*n* = 21/A:13.45 ± 1.12) CG (*n* = 18/A:11.27 ± 1.19)	IG_1_ = TB IG_2_ = FRD IG_3_ = Control	T_1_ = Pretreatment T_2_ = TB* the active treatment period was complete where there was no difference with and without the appliance. FDR*class II occlusion was‐overcorrected to an edge‐to‐edge incisal relationship	Skeletal Dento‐alveolar Pharyngeal airway	The twin block functional appliance induced significant skeletal and pharyngeal airway change compared to the effect induced by FFRD or by Natural growth.
Gulec and Goymen (2018). CCT, 2018, Turkey	Treatment of Class II Malocclusion: A Comparative Study of the Effects of Twin‐block and Fatigue Resistant Device	− 40 patients (TB:20/FDR: 20) − growing patients − 40 Patients (TB: 15‐FDR: 15‐CG:10). ‐(TB: 8 F‐7M/A:12.18 ± 2.19).(FDR: 8 F‐7M/‐A:12.9 ± 2.19).‐(CG:7F‐3M/A:12.77 ± 1.62	IG_1_: Twin block IG_2_: Forsus IG_3_: control	T_2_: In FRD group, after remove FDR. T_2_: In twin block after remove Twin‐block	Skeletal dental soft tissue	Both appliances were effective in correction class II‐Dental and skeletal Change‐enhance mandibular growth‐change soft tissue. But only TB restricts maxillary growth

### 3.3 Quantitative Synthesis of the Results

Data synthesis on the skeletal outcomes was not feasible to be conducted robustly due to the highly heterogeneous nature of the data. However, the pooled estimate revealed a statistically significant 1.3° increase in the SNB angle for patients treated with the TBA appliance compared with the FRD group. Additionally, a significant decrease of −1.34° in the ANB angle was observed in patients treated with TBA compared with the FRD group. Conversely, there were no statistically significant differences in the SNA angle between the two groups.

ANB: (MD = −1.34; 95% CI (1.03, 1.65); *p* < 0.00001). Heterogeneity was high (*χ* ^2^ = 48.08; *p* < 0.00001; I2 = 92%).

SNA:(MD = −0.62; 95% CI (−1.27, 0.03); *p* = 0.06). Heterogeneity was high (*χ* ^2^ = 10.02; *p* = 0.04; I2 = 60%).

SNB:(MD = 1.32; 95% CI (−1.67, −0.97); *p* < 0.00001). Heterogeneity was high (*χ* ^2^ = 28.04; *p* < 0.00001; I2 = 86%).

### 3.4 Risk of Bias Within Studies

**Figure 2:**
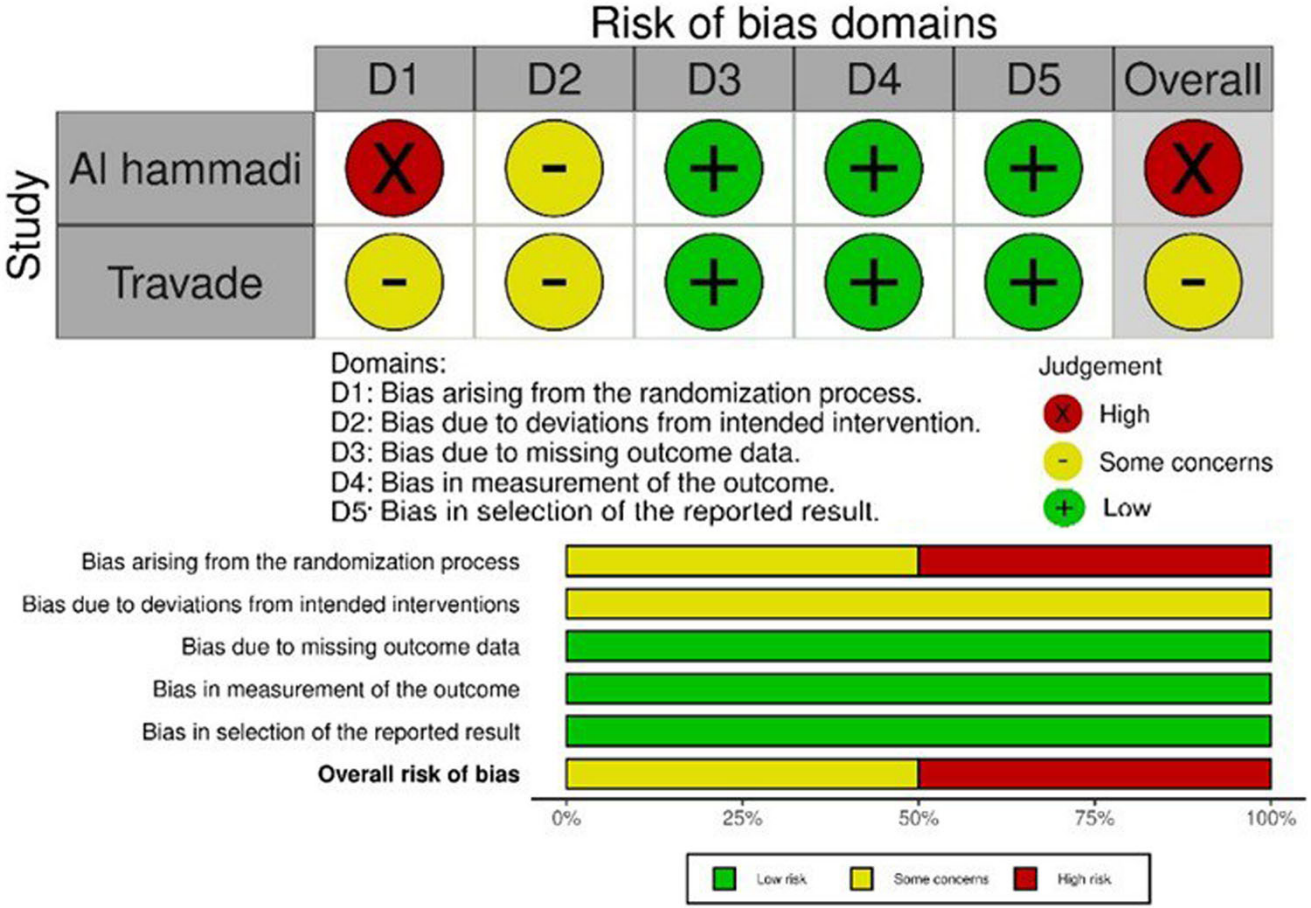
Summary of the risk of bias of randomized studies using ROB2 tool.

**Figure 3:**
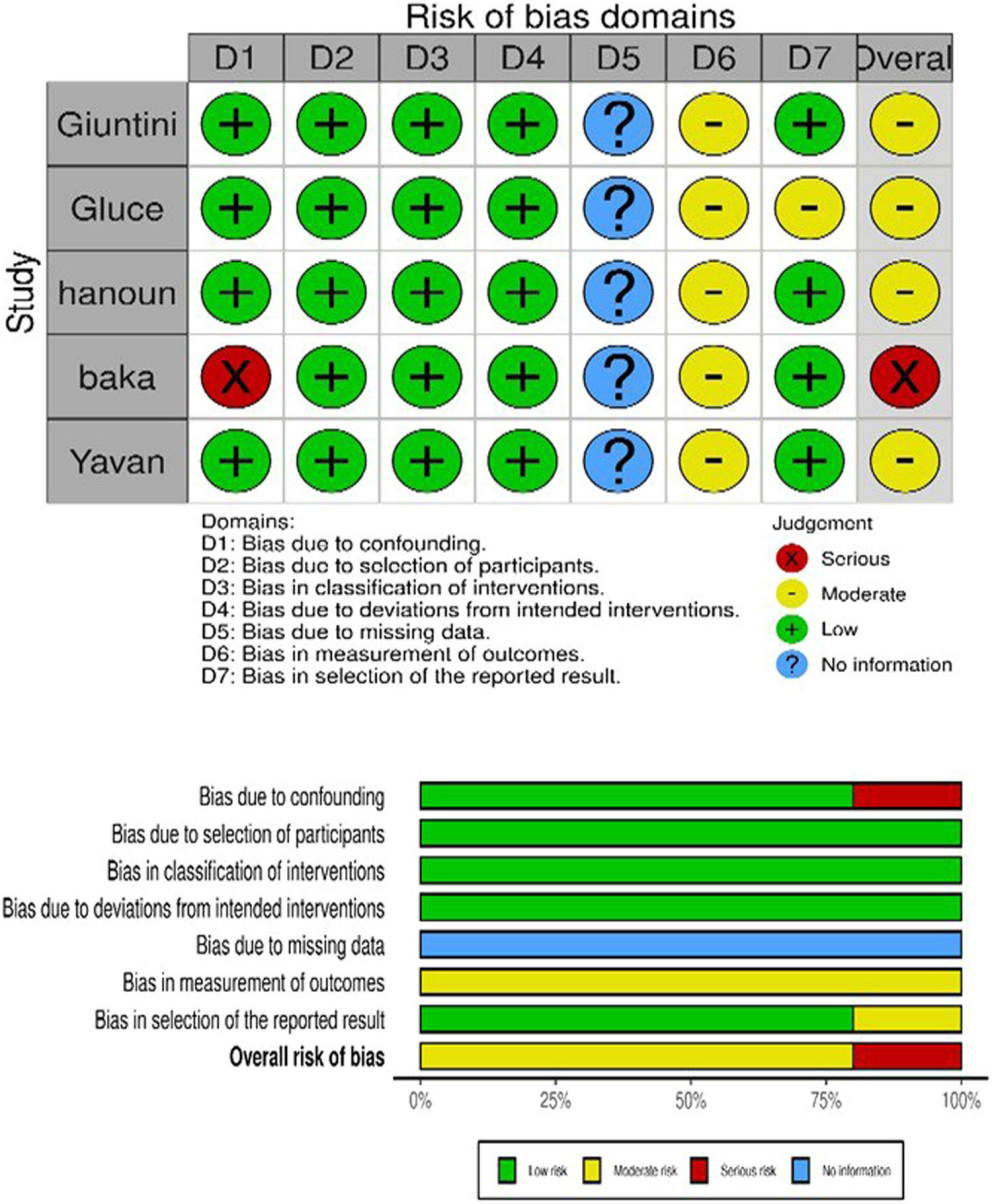
Summary of the risk of bias of non‐randomized studies using ROBINS‐I tool.

A summary of the overall RoB assessment applied to the included studies is provided in Figures 2 and 3. One RCT was evaluated to be at a moderate level and the other one was assessed to lurk at a high “risk degree” of RoB (Alhammadi et al. 2019; Tarvade et al. 2014). Operationalizing the ROBINS‐I tool onto the non‐RCTs, four of the five studies showed a moderate level of RoB; only non‐RCT was detected to be at serious risk of bias. More details regarding this assessment are displayed in Tables S2 and S3.

### 3.5 Effects of Interventions

All seven studies assessed the treatment effect of using FRD compared with TBA. Three studies (Giuntini et al. 2015; Gulec and Goymen 2018; Hanoun et al. 2014) assessed the treatment effect of utilizing FRD compared with TBA; they focused mainly on the skeletal and dentoalveolar structure outcomes. On the other hand, the remaining four studies (Baka and Fidanboy 2021; Yavan et al. 2021; Alhammadi et al. 2019; Tarvade et al. 2014) measured the pharyngeal airway and uvula‐glossopharyngeal changes as their main outcomes. A secondary outcome was the dentoskeletal changes and how they were influenced by the use of TBA or FRD as orthodontic treatment devices for correcting class II malocclusion.

*Skeletal changes:* Six studies (Baka and Fidanboy 2021; Giuntini et al. 2015; Gulec and Goymen 2018; Yavan et al. 2021; Alhammadi et al. 2019; Tarvade et al. 2014) comprising 254 patients for their population assessed the change in the following cephalometric angles: SNA, SNB, and ANB. Findings between the TBA and FRD groups were distributed as follows.

In connection to the SNA values, one study (Giuntini et al. 2015) showed a statistically significant decrease in the corresponding SNA values for patient outcomes in FRD compared with TBA [MD 1.10 and *p* < 0.005]. However, five studies concluded that there existed no significant differences in the SNA values between the two compared groups (Baka and Fidanboy 2021; Gulec and Goymen 2018; Yavan et al. 2021; Alhammadi et al. 2019; Tarvade et al. 2014).

When the focus is dedicated toward the SNB angle, three studies (Giuntini et al. 2015; Yavan et al. 2021; Alhammadi et al. 2019) determined that the TBA group manifested greater advancement of the SNB angle in comparison with the FRD participants. The mean differences were 1.88, 1.90, and 1.24, respectively. The *p*‐values were equal to 0.01, 0.01, and 0.045, respectively, across these three studies. Three other studies, nevertheless, presented no significant differences between the two groups (Baka and Fidanboy 2021; Gulec and Goymen 2018; Tarvade et al. 2014). Concerning the last ANB angle, three studies (Giuntini et al. 2015; Alhammadi et al. 2019; Tarvade et al. 2014) established a decrease in the ANB angle significantly apparent in the TBA groups. Mean differences were 0.80, −2.46, and −0.75, respectively, and *p*‐value was 0.01, 0.05, and 0.028. However, three studies showed no statistically significant differences between the TBA and FRD groups (Baka and Fidanboy 2021; Gulec and Goymen 2018; Yavan et al. 2021).

The mandibular length, at the cephalometric points of Gonion (Go) and Gnathion (Gn), was a decisive aspect of the outcome. The Go‐Gn mandibular linear measurement in (mm) was registered in the assessment of treatment consequences. Five studies handled this variable. Only two studies (Giuntini et al. 2015; Hanoun et al. 2014) concluded that the TBA showed a greater mandibular length, with mean differences to be 2.00 and 4.65, at *p* = 0.005 and 0.001, respectively. On the other hand, the other three studies (Gulec and Goymen 2018; Yavan et al. 2021; Tarvade et al. 2014) reported no significant differences between the TBA and FRD groups, under the consideration of mandibular length.

*Dentoalveolar changes:* The angle between the long axis of the lower incisor and mandibular plane L_1_/ML was used to assess the lower incisor inclination. Five studies addressed this angle. The results from three of these studies revealed a statically insignificant protrusion in the lower incisors, whereas two studies reported a significant protrusion between two groups, the FRD (Giuntini et al. 2015; Tarvade et al. 2014). In these two studies, MD were 2.90 and 9.33, respectively.

As for the U_1_ inclination, there are several angles referring to the inclination of the upper incisors. Some studies took the U_1_/SN angle as a point of measurement, while others took the Max1‐FH angle. Of the researched studies, only five studies reported these angles. Two studies (Giuntini et al. 2015; Hanoun et al. 2014) showed statically significant retraction in the maxillary incisors in the TBA group compared with the FRD group. Their mean differences were −6.50 and −8.5, respectively, at *p* < 0.001 in both of them. Nonetheless, three studies showed statically no significant differences between the TBA and FRD groups (Gulec and Goymen 2018; Yavan et al. 2021; Tarvade et al. 2014).

## 4 Discussion

One important goal of functional therapy for class II malocclusion is to correct mandibular retrognathia (DiBiase, Cobourne, and Lee 2015). Taking advantage of the positive skeletal effects and minimizing the dentoalveolar effects when using orthodontic appliances. Many devices and appliances have been used to achieve these results (Ngan and Tai 2023; Antonarakis and Kiliaridis 2007). Furthermore, the most important factor contributing to the success of functional treatment is the level of patient maturation (Khoja, Fida, and Shaikh 2016; King et al. 1990). Numerous studies have compared the effectiveness of treatment devices, namely TBA and FRD, in the early and late pubertal growth spurt periods. Baccetti et al. Pancherz, and O'Brien inferred that the major skeletal changes appeared more clearly in the late pubertal‐period group in their study samples. Yet, the early pubertal‐period groups admitted more notably distinguished effects concerning dentoalveolar alterations (Baccetti et al. 2000; O'Brien et al. 2003; Hansen, Pancherz, and Hagg 1991; Pancherz and Fackel 1990). A mere close review of the results on the SNA angle across studies is conflicting. Some show a decrease in SNA angle (Dada et al. 2015; Mahamad et al. 2012) others have found no significant restriction of maxillary growth (Khoja, Fida, and Shaikh 2016). Most of the studies showed that the SNA angle decreased in all research samples with no significant difference between the groups. However, only one study exposed a significant decrease in the SNA values in the FRD group, the “headgear effect.” First, the change in the SNA values may be attributed to a change in the incisor root apex rendering a noticeable retraction of Point‐A (Dada et al. 2015; Mahamad et al. 2012). In some studies, these results can be attributed to gaining more benefits from the growth spurt and rendering noticeably more adequate bone response on the part of the FRD group.

Another strikingly obvious treatment outcome relates to the SNB angle, as most of the studies showed a greater advancement in this angle for the TBA group. This came in accordance with what had been found throughout the literature (Khoja, Fida, and Shaikh 2016; Radwan, Maher, and Montasser 2022). This gives more prominence to the TBA treatment mode in posturing the mandible anterior than the ability of the FRD in such cases. This is ascribed to the difference in anchorage designs and the replacement of condyle position. However, three studies found no significant difference between the two groups. These discrepancies in results may also be attributed to variations in the ages of the participants across the treatment groups. The majority of the examined studies agreed that the TBA treatment mode offers a greater mandibular length than that offered by the FRD (Khoja, Fida, and Shaikh 2016). This may be due to higher overjet values in the TBA group at baseline interval, allowing for more stimulation of mandible growth. However, three studies found no difference between the two groups. Possible reasons include bias in data collection.

However, if dentoalveolar changes are highlighted now in the current systematic review, a number of considerations necessitate closer scrutinization. To begin with, the inclination of lower incisors is an important factor in orthodontic treatment (Tweed 1954). In fact, a number of factors can contribute to proclination of lower incisors regarding the TBA. The appliance design is an influencing factor where it can be manufactured with components to prevent flaring of lower incisors, such as capping the lower incisors with acryl or including forms of retention, like a ball‐ended clasp‐labial bow. Conversely, some studies affirm that the inclination of lower incisors occurs even with these methods of retention (Mills and McCulloch 1998; Jena, Duggal, and Parkash 2006; Ehsani et al. 2015). Some of the contacted studies confirmed the retroclination of upper incisors when they utilize the anterior upper bow [howlay wire] (Harradine and Gale 2000; Parkin, McKeown, and Sandler 2001). The majority of the studies showed that there is no difference concerning the retroclination of incisors whether when using the TBA or the FRD except for two studies. This may be attributed to specific design features and the timing of taking T_1_ records in trails. With reference to the upper incisors, the studies show that the FRD has a retroclination effect on the upper incisors, while the (TBA) group has revealed more retroclination of upper incisors in most studies (Aslan et al. 2014; Bilgiç, Başaran, and Hamamci 2015; Gunay, Arun, and Nalbantgil 2011; Oztoprak et al. 2012; Karacay et al. 2006). Clinically, the two appliances, TBA and FRD, possess the ability to reach the same point in terms of overjet, this result is in accordance with a previous study (Radwan, Maher, and Montasser 2022; Gunay, Arun, and Nalbantgil 2011; Oztoprak et al. 2012; Karacay et al. 2006; Heinrichs et al. 2014). However, the difference abides in the way leading to such a state; while the FRD renders more dentoalveolar effects, the TBA provides better skeletal effects, especially concerning mandibular advancement and mandibular length. This returns to the nature of the appliance and its work—heavy forces in intermittent times—along with its anchorage design.

## 5 Limitations

Low number of prospective studies and statistical heterogeneity across the collected data, as this prevented the possibility of conducting a meta‐analysis.

## 6 Conclusion

According to the available evidence, the FRD has been shown to be an effective treatment for correcting Class II skeletal malocclusion and excessive overjet. This is accomplished through an increase in both the SNB angle and length of the mandible, the restriction of point A, and the flaring of lower incisors, which was similar to those achieved through the use of the Twin Block. However, this result was less than achieved by using the TBA. Most of the studies presented a high bias and lack of blinding process because most studies were non‐RCTs. Consequently, we need more prospective, controlled clinical trials with larger sample sizes and uniform participant age to reach generalizable results.

## Author Contributions

Bahaa Aldeen Jeha and Rania Haddad conducted the electronic search, assessed full‐text articles, selected the studies, extracted data, and evaluated the risk of bias. They also wrote the initial draft of the manuscript. An arbiter assisted in resolving any disagreements that arose during the risk of bias assessment. Bahaa Aldeen Jeha and Rania Haddad developed the review question and participated in the writing stage. Finally, all authors approved the final version of this manuscript.

## Conflicts of Interest

The authors declare no conflicts of interest.

## Supporting information

Supplementary Table 1. Search strategy.

Supplementary Table 2. Details of the risk of bias of randomized studies.

Supplementary Table 3. Details of the risk of bias of non‐randomized studies.

## Data Availability

Data sharing is not applicable to this article as no new data were created or analyzed in this study.
