# Supplementary material for: Skeletal and Dental Effects of Forsus Fatigue Resistance Device Versus Twin Block Appliance for Class II Malocclusion Treatment in Growing Patients: A Systematic Review
Source: Clin Exp Dent Res. 2024 Dec 12;10(6):e70054. doi: 10.1002/cre2.70054 (PMC11636309; doi:10.1002/cre2.70054)
Supplement: Supplementary file 1 — Supplementary Table 1. Search strategy. [file CRE2-10-e70054-s002.docx]

|  | **Databases** | **Search Strategy** | **Results** |
| --- | --- | --- | --- |
| **1** | **CENTRAL (The Cochrane Library)**  From inception up to  22-7-2022  with no limits | #1 forsus OR "fixed functional appliance" OR "fixed functional treatment" OR "fatigue resistance device" OR ffrd  #2 Twin block* OR "Twin block Appliance" OR "Removable orthodontic Appliance" OR "Removable Appliance OR "Functional Treatment  #3 "class II" OR "class II malocclusion" OR "class II treatment" OR "class II correction" OR growth OR "skeletal class II"  #4 #1 AND #2 AND #3 | **#4= 10** |
| **2** | **Scopus**  Title-Abstract-Keyword  From inception up to  22-7-2022  with no limits | #1 forsus OR "fixed functional appliance" OR "fixed functional treatment" OR "fatigue resistance device" OR ffrd  #2 Twin block* OR "Twin block Appliance" OR "Removable orthodontic Appliance" OR "Removable Appliance OR "Functional Treatment  #3 "class II" OR "class II malocclusion" OR "class II treatment" OR "class II correction" OR growth OR "skeletal class II"  #4 #1 AND #2 AND #3 | **#4= 31** |
| **3** | **Web of Science**  **All Data Bases**  TS=Topics  From inception up to  22-7-2022  with no limits | #1 TS= (forsus OR "fixed functional appliance" OR "fixed functional treatment" OR "fatigue resistance device" OR ffrd)  #2 TS= Twin block* OR "Twin block Appliance" OR "Removable orthodontic Appliance" OR "Removable Appliance OR "Functional Treatment  #3 TS= ("class II" OR "class II malocclusion" OR "class II treatment" OR "class II correction" OR growth OR "skeletal class II")  #4 #1 AND #2 AND #3 | **#4= 26** |
| **4** | **EMBASE via OVID**  Keyword  From inception up to  22-7-2022  with no limits | #1 forsus OR "fixed functional appliance" OR "fixed functional treatment" OR "fatigue resistance device" OR ffrd  #2 Twin block* OR "Twin block Appliance" OR "Removable orthodontic Appliance" OR "Removable Appliance OR "Functional Treatment *  #3 "class II" OR "class II malocclusion" OR "class II treatment" OR "class II correction" OR growth OR "skeletal class II"  #4 #1 AND #2 AND #3 | **#4= 22** |
| **5** | **PubMed**  **All fields**  From inception up to  22-7-2022  with no limits | #1 forsus OR "fixed functional appliance" OR "fixed functional treatment" OR "fatigue resistance device" OR ffrd  #2Twin block* OR "Twin block Appliance" OR "Removable orthodontic Appliance" OR "Removable Appliance OR "Functional Treatment  #3 "class II" OR "class II malocclusion" OR "class II treatment" OR "class II correction" OR growth OR "skeletal class II"  #4 #1 AND #2 AND #3 | **#4= 47** |
|  |  | #1 Forsus | **139** |
|  |  | #2 Fixed functional appliance | **798** |
|  |  | #3 Fixed functional treatment | **5651** |
|  |  | #4 Fatigue resistance device | **207** |
|  |  | #1 OR #2 OR #3 OR #4 | **6543** |
|  |  | #7 Twin block | **129** |
|  |  | #8 Twin Block Appliance | **42** |
|  |  | #9 Removable orthodontic Appliance | **617** |
|  |  | #10 Removable Appliance | **595** |
|  |  | #7 OR #8 OR #9 OR #10 | **929** |
|  |  | #11 Class II | **12.778** |
|  |  | # 12 Class II malocclusion | **896** |
|  |  | #13 Class II treatment | **5.267** |
|  |  | #14 Class II correction | **571** |
|  |  | #15 growth | **288.311** |
|  |  | #16 Skeletal class II | **401** |
|  |  | #17 dental class II | **900** |
|  |  | #11 OR #12 OR #13 OR #14 OR #15 OR #16 | **299811** |
|  |  | #1 OR #2 OR #3 OR #4 AND #7 OR #8 OR #9 OR #10 AND #11 OR #12 OR #13 OR #14 OR #15 OR #16 OR #17 | **47** |
| **6** | **ClinicalTrials**  Advanced Search  Title  29-11-2020 | (forsus OR "fixed functional appliance" OR "fixed functional treatment" OR "fatigue resistance device" OR frd)  AND (Twin block* OR "Twin block Appliance" OR "Removable orthodontic Appliance" OR "Removable Appliance OR "Functional Treatment ) | **1** |
