# Supplementary material for: Skeletal and Dental Effects of Forsus Fatigue Resistance Device Versus Twin Block Appliance for Class II Malocclusion Treatment in Growing Patients: A Systematic Review
Source: Clin Exp Dent Res. 2024 Dec 12;10(6):e70054. doi: 10.1002/cre2.70054 (PMC11636309; doi:10.1002/cre2.70054)
Supplement: Supplementary file 2 — Supplementary Table 2. Details of the risk of bias of randomized studies. [file CRE2-10-e70054-s003.docx]

| **Study** | **Randomization bias** | **Effect of assignment to intervention** | **Missing data bias** | **Outcome measurement** | **Selection of the reported result** | **Overall** |
| --- | --- | --- | --- | --- | --- | --- |
| **Alhammadi et al.2019** | **1.1: NO Information**  “the researchers did not mention any method of randomization | **2.1: Yes**  “Blinding of the clinicians and patients to the interventions in each group was impossible”. | **3.1: NO**  As stated on page 6:  “some patients dropped out during the trial.” | **4.1: No**  Cephalometric measurements were used to assessment the outcomes which have a high validity for assessing anterior-posterior skeletal and dental changes | **5.1: P Y**  The trial was not registered, so the statistical analysis plan was compared with the results. | **Some concerns** |
|  | **1.2: No information**  The researchers did not provide any information about method of allocation | **2.2: Yes**  “Blinding of the clinicians and patients to the interventions in each group was impossible”. | **3.2: Yes**  The researcher have increased simple size to avoid the risk of withdrawal | **4.2: No**  The measurements were taken after treatment for both groups. | **5.2: P No**  The trial was not registered, so the statistical analysis plan was compared with the results.  All eligible reported results for the outcome domain correspond to all intended outcome measurements. |  |
|  | **1.3: YES**  Different mean age, ANB angle at baseline. | **2.3: No**  There were no deviations from the intended intervention that arose because of the trial context  (i.e., the treatment is unlikely to be influenced by lack of blinding) | **3.3: N/A**  (As the answer of 3.1 was Yes) | **4.3: P YES**  “Blinding is impossible for outcomes assessment because it contains determined reference points”. | **5.3: P No**  The trial was not registered, so the statistical analysis plan was compared with the results.  All eligible reported results for the outcome measurement correspond to all intended analyses. |  |
|  |  | **2.4: N/A**  (As the answer of 2.3 was No) | **3.4: N/A**  (As the answer of 3.1 was Yes) | **4.4: No**  (No the assessment of outcome was not influenced by knowledge of assessors) |  |  |
|  |  |  |  | **4.5: N/A**  (As the answer of 4.4 was No) |  |  |
|  | **Judgment: High** | **Judgment: Some concerns** | **Judgment: Low risk** | **Judgment: Low risk** | **Judgment:** **Low risk** |  |

| **Study** | **Randomization bias** | **Effect of assignment to intervention** | **Missing data bias** | **Outcome measurement** | **Selection of the reported result** | **Overall** |
| --- | --- | --- | --- | --- | --- | --- |
| **Travade et al.2013** | **1.1: NO Information**  “the researchers did not mention any method of randomization | **2.1: Yes**  The participants were aware of their assigned group as the treatment involved a two different appliance | **3.1: Yes**  : “None of the treatment groups’ patients discontinued the trial”. | **4.1: No**  The cephalo metric were used to assess the outcomes which have a high validity for assessing anterior-posterior skeletal and dental changes | **5.1: P Yes**  The trial was not registered, so the statistical analysis plan was compared with the results.  No changes to analysis plans were made before unblinded outcome data were available. | **Some concerns** |
|  | **1.2: No information**  The researchers did not provide any information about method of allocation | **2.2: Yes**  The carers were aware of the assigned group | **3.2: N/A**  (As the answer of 3.1 was Yes) | **4.2: No**  The measurement were taken before and after treatment for both groups | **5.2: P No**  The trial was not registered, so the statistical analysis plan was compared with the results.  All eligible reported results for the outcome domain correspond to all intended outcome measurements. |  |
|  | **1.3: No information**  No information about comparison between group at baseline . | **2.3: No**  There were no deviations from the intended intervention that arose because of the trial context  (i.e. the treatment is unlikely to be influenced by lack of blinding) | **3.3: N/A**  (As the answer of 3.1 was Yes) | **4.3: Yes**  Blinding is impossible for outcomes assessment because it contains determined reference points -No information about assessor blinding to analysis | **5.3: P No**  The trial was not registered, so the statistical analysis plan was compared with the results.  All eligible reported results for the outcome measurement correspond to all intended analyses. |  |
|  |  | **2.4: N/A**  (As the answer of 2.3 was No) | **3.4: N/A**  (As the answer of 3.1 was Yes) | **4.4: N/A**  (No the assessment of outcome was not influenced by knowledge of assessors) |  |  |
|  |  |  |  | **4.5: N/A**  (As the answer of 4.3 was No) |  |  |
|  | **Judgment: Some concerns** | **Judgment: Some concerns** | **Judgment: Low risk** | **Judgment: Low risk** | **Judgment: Low risk** |  |
