# Supplementary material for: Skeletal and Dental Effects of Forsus Fatigue Resistance Device Versus Twin Block Appliance for Class II Malocclusion Treatment in Growing Patients: A Systematic Review
Source: Clin Exp Dent Res. 2024 Dec 12;10(6):e70054. doi: 10.1002/cre2.70054 (PMC11636309; doi:10.1002/cre2.70054)
Supplement: Supplementary file 3 — Supplementary Table 3. Details of the risk of bias of non‐randomized studies. [file CRE2-10-e70054-s001.docx]

| **Study** | **Pre-intervention** | | **At intervention** | **Post-intervention** | | | | **Overall** |
| --- | --- | --- | --- | --- | --- | --- | --- | --- |
|  | **Confounding** | **Selection of participants** | **Classification bias** | **Deviation from intended intervention** | **Missing data** | **Outcome measurement** | **Bias in the selection of the result** |  |
| **Giuntini et al. 2015** | **1.1** **NO**  According to the characteristics at baseline, there are a no differences between groups except for the overbite, which could not be considered as a confounding factor for functional treatments. | **2.1** **P No**  The design of the study involved either applying a twin-block appliance treatment or (FDR) and Hence, it is unlikely to select the participants based on the characteristics observed after the start of intervention. | **3.1 yes**  Authors mentioned Detailed information about methods and treatment protocols ''As on page 2'' | **4.1** **No**  There were no information deviations from the intended intervention beyond what would be expected in usual practice | **5.1**  **NO Information**  There is no Information  about missing data was reported.. | **6.1** **Yes**  As the outcome measure involve manual determination of the cephalogram points | **7.1** **No**  All possible measurements to determine the effects were reported. | **Moderate Risk of bias** |
|  | **1.2 No**  The analysis based on splitting participants’ follow up time according to intervention received was not applied. |  |  |  |  |  |  |  |
|  | **1.3 N/A**  (As the answer of 1.2 was No**)** | **2.2 N/A**  (As the answer of 2.1 was P No**)** | **3.2 Yes**  Misclassification of intervention status is unlikely. غير ممكن))  (Data were collected through cephalograms). | **4.2 N/A**  (As the answer of 4.1 was No**)** | **5.2**  **NO Information**  There is no Information  about missing data was reported. | **6.2** **NO Information**  There is no Information about assessor | **7.2 No**  The analyst pre-specified the methods to be applied |  |
|  | **1.4 Yes**  Restricting eligibility criteria were used to individuals, who all have the same value at the baseline. |  |  | **4.3** **Yes**  No co-interventions across intervention groups were applied. |  |  |  |  |
|  | **1.5 Yes**  The used variables are not subjective | **2.3 N/A**  (As the answer of 2.1 was P No**)** | **3.3** **No**  Misclassification of intervention status is unlikely.  (Data were collected through cephalograms). | **4.4** **YES**  Implementation of the intervention was successful for most participants | **5.3**  **NO Information**  There is no Information  about missing data was reported**.** | **6.3** YES  Same time point, same definition,  and same measurements were applied for both groups |  |  |
|  | **1.6 P No**  Post-intervention variables were assessed by cephalograms, so it is unlikely that authors can control these variables during treatment. |  |  |  |  |  |  |  |
|  | **1.7 N/A**  (As the answer of 1.2 was No**)** | **2.4 Yes**  Participants were followed from the start of the intervention |  | **4.5** **YES**  The study was Retrospective study Contain applied fixed appliance and removable one, impairment adherence and cross overs to comparator is not possible. | **5.4**  (As the answer of 5.1 was NO**)** | **6.4 No**  No systematic errors in measuring the outcome were reported | **7.3** **P No**  The sample is not considered as large cohorts. |  |
|  | **1.8 N/A**  (As the answer of 1.2 was No**)** | **2.5** **N/A**  (As the answer of 2.4 was Yes**)** |  | **4.6**  (As the answer of 4.5 was Yes**)** | **5.5** (As the answer of 5.1 was NO**)** |  |  |  |
|  | **Low risk** | **Low risk** | **Low risk** | **Low risk** | **No Information** | **Moderate Risk of bias** | **Low risk** |  |

| **Study** | **Pre-intervention** | | **At intervention** | **Post-intervention** | | | | **Overall** |
| --- | --- | --- | --- | --- | --- | --- | --- | --- |
|  | **Confounding** | **Selection of participants** | **Classification bias** | **Deviation from intended intervention** | **Missing data** | **Outcome measurement** | **Bias in the selection of the result** |  |
| **Gulec et al. 2018** | **1.1** **NO**  According to the characteristics at baseline, there are NO differences between groups expect soft tissues which could not be considered as a confounding factor for functional treatments | **2.1** **P No**  The design of the study involved either applying a twin-block appliance treatment or (FDR) or not applying any appliance. Hence, it is unlikely to select the participants based on the characteristics observed after the start of intervention. | **3.1 Yes**  Authors mentioned Detailed information about methods and treatment protocols | **4.1** **No**  There were no information deviations from the intended intervention beyond what would be expected in usual practice | **5.1**  **NO Information**  There is no Information  about missing data was reported. | **6.1** **Yes**  As the outcome measure involve manual determination of the cephalogram points | **7.1** **No**  All possible measurements to determine the effects were reported. | **Moderate Risk of bias** |
|  | **1.2 No**  The analysis based on splitting participants’ follow up time according to intervention received was not applied. |  |  |  |  |  |  |  |
|  | **1.3 N/A**  (As the answer of 1.2 was No**)** | **2.2 N/A**  (As the answer of 2.1 was P No**)** | **3.2 Yes**  Misclassification of intervention status is unlikely.  (Data were collected through cephalogram). | **4.2 N/A**  (As the answer of 4.1 was No**)** | **5.2**  **NO Information**  There is no Information  about missing data was reported. | **6.2** **No information**  No information was reported about the outcome assessor | **7.2 No**  The analyst pre-specified the methods to be applied |  |
|  | **1.4 Yes**  Restricting eligibility criteria were used to individuals, who all have the same value at the baseline. |  |  | **4.3** **Yes**  No co-interventions across intervention groups were applied. |  |  |  |  |
|  | **1.5 Yes**  The used variables are not subjective | **2.3 N/A**  (As the answer of 2.1 was P No**)** | **3.3** **No**  Misclassification of intervention status is unlikely.  (Data were collected through cephalogram ). | **4.4** **YES**  Implementation of the intervention was successful for all participants | **5.3** **NO Information**  There is no Information  about missing data was reported. | **6.3** YES  Same time point, same definition,  and same measurements were applied for both groups |  |  |
|  | **1.6 P No**  Post-intervention variables were assessed by cephalogram, so it is unlikely that authors can control these variables during treatment. |  |  |  |  |  |  |  |
|  | **1.7 N/A**  (As the answer of 1.2 was No**)** | **2.4 YES**  ALL Participants were followed from the start of the intervention |  | **4.5** **YES**  The study is retrospective study applied fixed appliance and removable one, and comparied with control that imperfect adherence is not possible. | **5.4**  (As the answer of 5.1 was NO**)** | **6.4 No**  No systematic errors in measuring the outcome were reported | **7.3** **P No**  The sample is not considered as large cohorts. |  |
|  | **1.8 N/A**  (As the answer of 1.2 was No**)** | **2.5** **N/A**  (As the answer of 2.4 was Yes**)** |  | **4.6**  (As the answer of 4.5 was Yes**)** | **5.5** (As the answer of 5.1 was NO**)** |  |  |  |
|  | **Low risk** | **Low risk** | **Low risk** | **Low risk** | **NO Information** | **Moderate Risk of bias** | **Low risk** |  |

| **Study** | **Pre-intervention** | | **At intervention** | **Post-intervention** | | | | **Overall** |
| --- | --- | --- | --- | --- | --- | --- | --- | --- |
|  | **Confounding** | **Selection of participants** | **Classification bias** | **Deviation from intended intervention** | **Missing data** | **Outcome measurement** | **Bias in the selection of the result** |  |
| **Hanoun et al. 2014** | **1.1** **NO**  According to the characteristics at baseline, there are NO differences between groups expect some value would not be considering as confounding | **2.1** **P No**  The design of the study involved either applying a twin-block appliance treatment or (FDR) or not applying any appliance. Hence, it is unlikely to select the participants based on the characteristics observed after the start of intervention. | **3.1 Yes**  Authors mentioned Detailed information about methods and treatment protocols | **4.1** **No**  There were no information deviations from the intended intervention beyond what would be expected in usual practice | **5.1**  **NO Information**  There is no Information  about missing data was reported.. | **6.1** **Yes**  As the outcome measure involve manual determination in the cephalogram points | **7.1** **No**  All possible measurements to determine the effects were reported. | **Moderate Risk of bias** |
|  | **1.2 No**  The analysis based on splitting participants’ follow up time according to intervention received was not applied. |  |  |  |  |  |  |  |
|  | **1.3 N/A**  (As the answer of 1.2 was No**)** | **2.2 N/A**  (As the answer of 2.1 was P No**)** | **3.2 Yes**  Misclassification of intervention status is unlikely.  (Data were collected through cbct). | **4.2 N/A**  (As the answer of 4.1 was No**)** | **5.2**  **NO Information**  There is no Information  about missing data was reported. | **6.2** **No information**  No information was reported about the outcome assessor | **7.2 No**  The analyst pre-specified the methods to be applied |  |
|  | **1.4 Yes**  Restricting eligibility criteria were used to individuals, who all have the same value at the baseline. |  |  | **4.3** **Yes**  No co-interventions across intervention groups were applied. |  |  |  |  |
|  | **1.5 Yes**  The used variables are not subjective | **2.3 N/A**  (As the answer of 2.1 was P No**)** | **3.3** **No**  Misclassification of intervention status is unlikely.  (Data were collected through CBCT). | **4.4** **YES**  Implementation of the intervention was successful for most participants | **5.3** **NO Information**  There is no Information  about missing data was reported. | **6.3** YES  Same time point, same definition,  and same measurements were applied for both groups |  |  |
|  | **1.6 P No**  Post-intervention variables were assessed by cephalograms, so it is unlikely that authors can control these variables during treatment. |  |  |  |  |  |  |  |
|  | **1.7 N/A**  (As the answer of 1.2 was No**)** | **2.4 YES**  ALL Participants were followed from the start of the intervention |  | **4.5** **YES**  The study is retrospective applied fixed appliance and removable one, and comparied with control that imperfect adherence is not possible. | **5.4**  (As the answer of 5.1 was NO**)** | **6.4 No**  No systematic errors in measuring the outcome were reported | **7.3** **P No**  The sample is not considered as large cohorts. |  |
|  | **1.8 N/A**  (As the answer of 1.2 was No**)** | **2.5** **N/A**  (As the answer of 2.4 was Yes**)** |  | **4.6**  (As the answer of 4.5 was Yes**)** | **5.5** (As the answer of 5.1 was NO**)** |  |  |  |
|  | **Low risk** | **Low risk** | **Low risk** | **Low risk** | **NO Information**. | **Moderate Risk of bias** | **Low risk** |  |

| **Study** | **Pre-intervention** | | **At intervention** | **Post-intervention** | | | | **Overall** |
| --- | --- | --- | --- | --- | --- | --- | --- | --- |
|  | **Confounding** | **Selection of participants** | **Classification bias** | **Deviation from intended intervention** | **Missing data** | **Outcome measurement** | **Bias in the selection of the result** |  |
| **Zeliha baka et al. 2019** | **1.1** **Yes**  No comparison between groups was done at baseline to ensure that there are no significant differences among the participants in the groups | **2.1** **P No**  The design of the study involved either applying a twin-block appliance treatment or (FDR). Hence, it is unlikely to select the participants based on the characteristics observed after the start of intervention. | **3.1 Yes**  Authors mentioned Detailed information about methods and treatment protocols | **4.1** **No**  There were no information deviations from the intended intervention beyond what would be expected in usual practice | **5.1**  **NO Information**  There is no Information  about missing data was reported. | **6.1** **Yes**  As the outcome measure involve manual determination of the cephalogram points | **7.1** **No**  All possible measurements to determine the the goal of study were reported. | **Serious risk of bias** |
|  | **1.2 No**  The analysis based on splitting participants’ follow up time according to intervention received was not applied. |  |  |  |  |  |  |  |
|  | **1.3 N/A**  (As the answer of 1.2 was No**)** | **2.2 N/A**  (As the answer of 2.1 was P No**)** | **3.2 Yes**  Misclassification of intervention status is unlikely.  (Data were collected through cephalograms). | **4.2 N/A**  (As the answer of 4.1 was No**)** | **5.2**  **NO Information**  There is no Information  about missing data was reported.. | **6.2** **No information**  No information was reported about the outcome assessor | **7.2 No**  The analyst pre-specified the methods to be applied |  |
|  | **1.4 P NO**  Restricting eligibility criteria were used to individuals, however intergroup comparison was not done at the baseline. |  |  | **4.3** **Yes**  No co-interventions across intervention groups were applied. |  |  |  |  |
|  | **1.5 Yes**  The used variables are not subjective | **2.3 N/A**  (As the answer of 2.1 was P No**)** | **3.3** **No**  Misclassification of intervention status is unlikely.  (Data were collected through cephalograms). | **4.4** **YES**  Implementation of the intervention was successful for all participants | **5.3** **NO Information**  There is no Information  about missing data was reported. | **6.3** YES  Same time point, same definition,  and same measurements were applied for both groups |  |  |
|  | **1.6 P No**  Post-intervention variables were assessed by cephalograms, so it is unlikely that authors can control these variables during treatment. |  |  |  |  |  |  |  |
|  | **1.7 N/A**  (As the answer of 1.2 was No**)** | **2.4 Yes**  As retrospective study the records obtained belong to Participants were followed from the start to the end of the intervention |  | **4.5** **YES**  The study is retrospective applied fixed appliance and removable one that impairment adherence is not possible. | **5.4**  (As the answer of 5.1 was NO**)** | **6.4 No**  No systematic errors in measuring the outcome were reported | **7.3** **P No**  The sample is not considered as large cohorts. |  |
|  | **1.8 N/A**  (As the answer of 1.2 was No**)** | **2.5** **N/A**  (As the answer of 2.4 was Yes**)** |  | **4.6**  (As the answer of 4.5 was Yes**)** | **5.5** (As the answer of 5.1 was NO**)** |  |  |  |
|  | **Serious Risk of bias** | **Low risk** | **Low risk** | **Low risk** | **No Information** | **Moderate Risk of bias** | **Low risk** |  |

| **Study** | **Pre-intervention** | | **At intervention** | **Post-intervention** | | | | **Overall** |
| --- | --- | --- | --- | --- | --- | --- | --- | --- |
|  | **Confounding** | **Selection of participants** | **Classification bias** | **Deviation from intended intervention** | **Missing data** | **Outcome measurement** | **Bias in the selection of the result** |  |
| **Yavan et al. 2021** | **1.1** **NO**  According to the characteristics at baseline, there are NO differences between both groups | **2.1** **P No**  The design of the study involved either applying a twin-block appliance or (FDR). Hence, it is unlikely to select the participants based on the characteristics observed after the start of intervention. | **3.1 yes**  Authors mentioned Detailed information about methods and treatment protocols | **4.1** **No**  There were no information deviations from the intended intervention beyond what would be expected in usual practice | **5.1**  **NO Information**  There is no Information  about missing data was reported.. | **6.1** **Yes**  As the outcome measure involve manual determination of the cephalogram points | **7.1** **No**  All possible measurements to determine the effects were reported. | **Moderate Risk of bias** |
|  | **1.2 No**  The analysis based on splitting participants’ follow up time according to intervention received was not applied. |  |  |  |  |  |  |  |
|  | **1.3 N/A**  (As the answer of 1.2 was No**)** | **2.2 N/A**  (As the answer of 2.1 was P No**)** | **3.2 Yes**  Misclassification of intervention status is unlikely.  (Data were collected through cephalograms). | **4.2 N/A**  (As the answer of 4.1 was No**)** | **5.2**  **NO Information**  There is no Information  about missing data was reported.. | **6.2** **No information**  No information was reported about the outcome assessor | **7.2 No**  The analyst pre-specified the methods to be applied |  |
|  | **1.4 Yes**  Restricting eligibility criteria were used to individuals, who all have the same value at the baseline. |  |  | **4.3** **Yes**  No co-interventions across intervention groups were applied. |  |  |  |  |
|  | **1.5 Yes**  The used variables are not subjective | **2.3 N/A**  (As the answer of 2.1 was P No**)** | **3.3** **No**  Misclassification of intervention status is unlikely.  (Data were collected through cephalograms). | **4.4** **YES**  Implementation of the intervention was successful for all participants | **5.3**  **NO Information**  There is no Information  about missing data was reported.. | **6.3** YES  Same time point, same definition,  and same measurements were applied for both groups |  |  |
|  | **1.6 P No**  Post-intervention variables were assessed by cephalograms, so it is unlikely that authors can control these variables during treatment. |  |  |  |  |  |  |  |
|  | **1.7 N/A**  (As the answer of 1.2 was No**)** | **2.4 Yes**  As retrospective study the records obtained belong to Participants were followed from the start to the end of the intervention |  | **4.5** **YES**  The study is retrospective applied fixed appliance and removable one so that imperfect adherence is not possible. | **5.4**  (As the answer of 5.1 was NO**)** | **6.4 No**  No systematic errors in measuring the outcome were reported | **7.3** **P No**  The sample is not considered as large cohorts. |  |
|  | **1.8 N/A**  (As the answer of 1.2 was No**)** | **2.5** **N/A**  (As the answer of 2.4 was Yes**)** |  | **4.6**  (As the answer of 4.5 was Yes**)** | **5.5** (As the answer of 5.1 was NO**)** |  |  |  |
|  | **Low risk** | **Low risk** | **Low risk** | **Low risk** | **NO Information** | **Moderate Risk of bias** | **Low risk** |  |
